# Associations Between Person-Centered Primary Care Measure Score, Potential Case-Mix Adjustment Variables, and Other Patient Experience Survey Items

**DOI:** 10.1007/s11606-024-08791-x

**Published:** 2024-05-06

**Authors:** Mark W. Friedberg, Wei Ying, James Courtemanche, Barbra G. Rabson

**Affiliations:** 1 Blue Cross Blue Shield of Massachusetts, Boston, MA USA; 2 Harvard Medical School, Boston, USA; 3 Brigham and Women’s Hospital, Boston, USA; 4 Massachusetts Health Quality Partners, Brighton, MA USA

## INTRODUCTION

The Person-Centered Primary Care Measure (PCPCM) received National Quality Forum endorsement in 2021 as a patient-reported outcome performance measure.^1, 2^ Local stakeholders suggested that Blue Cross Blue Shield of Massachusetts (BCBSMA) use the PCPCM as a basis for pay-for-performance for primary care practitioners (PCPs). BCBSMA requires validity and reliability for financially incentivized measures.

Validity of patient survey measures hinges on adequate risk adjustment. The Clinician and Group Consumer Assessment of Healthcare Providers and Systems (CG-CAHPS) survey, for example, features case-mix adjustment methods.^3^ However, the PCPCM lacks case-mix adjustment methods. No published analyses have assessed whether the PCPCM requires case-mix adjustment.

## METHODS

We surveyed BCBSMA members in June–August 2021 in two mail-only waves, using a Massachusetts Health Quality Partners (MHQP) patient experience survey instrument based on the CG-CAHPS Visit Survey 4.0, with embedded PCPCM items.^4^

The survey sample included 2400 BCBSMA members who were attributed to PCPs (2000 to 20 physicians, 400 to 29 nurse practitioners) using HMO member self-designation or, in absence of self-designation (most PPO members), the PCP with most recent evaluation and management claim and who had an in-person or virtual primary care visit between September 1, 2020, and February 28, 2021.

We stratified the survey sample by length of relationship with attributed PCP (< 1 year, 1–4 years, > 4 years) and by medical complexity based on claims (high, medium, low; DxCG/Cotiviti).

For each returned complete survey, we calculated PCPCM scores (0 to 100), scores on two global ratings–based MHQP survey items—overall rating of most recent visit (0 to 10), likelihood to recommend provider (0 to 10)—and seven report-based MHQP composites (i.e., those assessing specific activities or events, all scored 0 to 100): Communication, Access, Integration of Care, Knowledge of Patient, Self-Management Support, Office Staff, and Adult Behavioral Health. Detailed information on these composites is available at MHQP.^5^

Using individual patient responses as units of analysis, we fit regression models that predicted each outcome score as a function of multiple potential case-mix adjustment variables (treating each as a linear predictor): self-identified age, gender, educational attainment, general health, mental health, self-identified length of relationship with PCP, whether most recent visit was in-person, claims-based medical complexity, and claims-based length of relationship with PCP. We also computed associations between PCPCM scores and the other outcome scores.

We used inverse probability of response weights to account for survey non-response and stratified sample design. We performed a complete case analysis, without imputing item-level non-responses. We considered *p*-values < 0.05 statistically significant.

This research was exempted from review by the WCG IRB.

## RESULTS

We received 381 survey responses (15.9% response rate). PCPCM scores were statistically significantly associated with self-reported general health, self-reported mental health, claims-based medical complexity, length of relationship with the PCP (both self-reported and claims-derived), and whether the most recent visit was in-person (Table 1). Overall rating of most recent visit was associated with self-reported general health. Likelihood to recommend provider was associated with self-reported general health, claims-based medical complexity, and whether the most recent visit was in-person. Of the report-based composites, only Knowledge of Patient was associated with more than one case-mix adjustment variable.

**Table 1:** Associations between Outcome Variables and Potential Case-Mix Adjustment Variables

Potential case-mix adjustment variable		PCPCM	Global ratings–based MHQP survey items		Report-based MHQP composites						
			Overall rating of most recent visit	Likelihood to recommend provider	Communication	Access	Integration of care	Knowledge of patient	Self-management support	Office staff	Adult behavioral health
Member age category (1–7; 1 = 18 to 24, 7 = 75 or older)	Coefficient estimate	− 0.95	− 0.60	− 1.08	− 0.83	− 2.03	− 1.85	− 0.64	− 2.68	0.14	− 4.04
	*p*-value	0.34	0.41	0.43	0.15	0.16	0.11	0.35	0.27	0.65	0.10
Member self-reported gender is male (1 = yes, 0 = no)	Coefficient estimate	0.32	2.75	4.37	2.04	1.76	4.63	0.75	5.70	− 0.59	− 12.33
	*p*-value	0.90	0.10	0.09	0.16	0.62	0.14	0.66	0.30	0.64	0.03
Education category, self-reported (1–6; 1 = 8th grade or less, 6 = more than 4-year college degree)	Coefficient estimate	1.84	1.47	1.75	0.54	− 2.53	0.52	0.79	0.87	0.55	3.25
	*p*-value	0.13	0.14	0.28	0.52	0.08	0.64	0.41	0.69	0.08	0.20
General health, self-reported (1–5; 1 = excellent, 5 = poor)	Coefficient estimate	− 3.80	− 4.07	− 5.69	− 1.07	− 2.76	− 0.04	− 2.20	− 1.47	− 0.93	− 1.35
	*p*-value	0.03	0.01	0.01	0.48	0.16	0.98	0.06	0.68	0.36	0.72
Mental health, self-reported (1–5; 1 = excellent, 5 = poor)	Coefficient estimate	− 5.49	− 3.70	− 4.08	− 2.89	0.16	− 1.63	− 4.38	− 9.79	− 1.38	− 4.18
	*p*-value	0.01	0.08	0.10	0.25	0.94	0.41	0.03	0.005	0.08	0.32
Medical complexity category, claims based (1 = less complex, 3 = more complex)	Coefficient estimate	4.44	2.37	5.38	0.21	− 0.56	− 0.13	1.15	8.29	− 0.51	4.59
	*p*-value	0.04	0.17	0.048	0.90	0.83	0.95	0.48	0.06	0.51	0.34
Relationship length with PCP, self-reported (1–5; 1 = less than 6 months, 5 = 5 years or more)	Coefficient estimate	3.61	1.22	1.51	1.26	2.81	− 0.12	2.07	1.52	0.002	1.10
	*p*-value	0.01	0.27	0.44	0.22	0.12	0.94	0.048	0.60	0.997	0.70
Relationship length with PCP, based on health plan administrative data (1 = less than 1 year, 3 = more than 4 years)	Coefficient estimate	3.25	0.87	2.47	0.002	1.06	1.37	2.11	6.77	0.97	− 5.43
	*p*-value	0.03	0.36	0.12	0.998	0.65	0.54	0.03	0.08	0.30	0.21
Most recent PCP visit was in person (1 = yes, 0 = no)	Coefficient estimate	− 8.62	− 3.58	− 7.24	− 4.21	13.27	− 4.77	− 1.92	− 3.10	3.3	11.32
	*p*-value	0.001	0.10	0.02	0.02	0.007	0.18	0.42	0.69	0.11	0.16

Coefficient estimates and *p*-values from SAS PROC GENMOD, weighted to account for non-response, with empirical standard error estimates, with outcome variables transformed to a 0- to 100-point scale

*Abbreviations*: *PCPCM* Person-Centered Primary Care Measure, *MHQP* Massachusetts Health Quality Partners, *PCP* primary care practitioner

PCPCM scores had Pearson’s correlations of 0.74 with overall visit rating, 0.72 with likelihood to recommend, 0.70 with Knowledge of Patient, and correlations < 0.6 with the remaining report-based composites (all statistically significant at *p* < 0.001).

## DISCUSSION

In a Massachusetts pilot, PCPCM scores were associated with standard CG-CAHPS case-mix adjustment variables, claims-based measures of medical complexity, and both claims-based and self-reported length of relationship with the rated PCP. The PCPCM was more highly associated with global ratings items (i.e., “satisfaction” items such as overall rating of most recent visit and likelihood to recommend provider) and Knowledge of Patient than with other report-based scores (e.g., Integration of Care).

The survey was limited to a single state, with modest sample size and response rate.

Our findings suggest that the PCPCM requires development of case-mix adjustment methods. Without case-mix adjustment, threats to validity include, for example, the potential to penalize early career PCPs and those who have recently moved, due to the significant association between length of relationship and PCPCM scores. Additionally, the PCPCM has little independence from existing global ratings survey items (e.g., likelihood to recommend provider).
